# Co-operative learning, psychometric adaptation, and invariability of the academic satisfaction scale in Spanish university students

**DOI:** 10.3389/fpsyg.2022.864510

**Published:** 2022-09-21

**Authors:** María Auxiliadora Robles-Bello, David Sánchez-Teruel, Óscar Gavin Chocano, Alfonso González Luque, José Antonio Camacho Conde

**Affiliations:** ^1^Faculty of Humanities and Education Sciences, University of Jaén, Jaén, Spain; ^2^Faculty of Psychology, University of Granada, Granada, Spain; ^3^Faculty of Sciences, National University of Distance Education (UNED), Madrid, Spain; ^4^Faculty of Education, Economy and Technology of Ceuta, University of Granada, Ceuta, Spain

**Keywords:** cooperative learning, academic satisfaction, Spanish population, invariance, psychometric properties

## Abstract

It is necessary to understand the measurement of academic satisfaction (AS) in a variety of cross-cultural contexts. The first aim was to evaluate the psychometric properties of AS scale, to explore its structural validity, to assess its differential item function, including gender and age invariance in university students. Study 2 aimed to assess whether AS improved after the application of a teaching instructional approach based on cooperative learning (CL), while a cross-sectional study was performed in several stages. Descriptive, confirmatory, and scale reliability analyses were carried out with indices for goodness-of-fit, such that a new scale was obtained with a single-factor structure. A reduction to 6-items in this sample exhibited better psychometric properties. Configural invariance by gender and age indicated that men and women had a similar understanding of the new scale. Given significant differences between groups, the CL group scored higher in AS.

## Introduction

Academic satisfaction (AS) is a dynamic process, influenced by the characteristics of the educational institution and by how students perceive their learning environment (Ramos et al., 2015). It has been studied in the university setting with a variety of conceptual approaches, based on quality of service and/or psychological wellbeing (Shin and Jung, 2014). From this perspective, satisfaction refers to students’ comparison of their aspirations with their achievements (Medrano et al., 2014). These assessments may be made considering our general lives and specific domains, including the academic experience (Osorio-Alvarez and Parra, 2015). According to Medrano et al. (2014), satisfaction in academia is seen as the enjoyment students have with experiences linked to their role. For Insunza et al. (2015), it also refers to students’ favorable subjective assessments of their education-related experiences and results. As such, AS can be considered a cognitive–affective variable, involving students’ excitement with the learning process and evaluating their experiences. It is a key influence in the adaptation to the academic environment (Righi et al., 2006), social integration (Medrano and Pérez, 2010), consistency in academic performance (Merino-Soto et al., 2016), and psychological wellbeing (Abarca et al., 2013); this construct is linked to positive mental health (Lukat et al., 2016), which contributes to reducing the negative effects with unpleasant experiences while maintaining an adaptive response to stressful situations (Iasiello et al., 2019). At present, there are few evaluation instruments for AS, although different studies corroborate the positive relationship with life satisfaction through the *Satisfaction with Life Scale* (SWLS) Instrument (Diener et al., 1985). Based on this approach, linked to wellbeing and positive feelings (Muñoz-Campos et al., 2018), it is fundamental to an individual’s aspirations, where variables such as self-efficacy (SE), a strategic resource that favors study and learning, and emotional affects attributed to success or failure, which have a direct impact on academic activity (Fiori and Vesely-Maillefer, 2018). Life satisfaction for students is geared to assess why those with the same skills present different behaviors and results (Feldman and Kubota, 2015).

The AS of university students has become vital for institutions in recent decades, which is conditioned by how students are a guaranteed factor in educational organizations (Huebner and Gilman, 2006; Lodi et al., 2017). Some studies find that the result is the sum of the student’s academic, emotional, and social experiences, with perceptions and expectations evolving (Méndez-Vera and Gálvez-Nieto, 2018). AS is a multidimensional and complex phenomenon, which acquires value along with other variables, such as emotional intelligence (EI) (Ferrero et al., 2021). The AS Scale from Lent et al. (2005) uses 7 items with a 5-point Likert scale; they form a single factor that measure perceptions of enjoyment as well as the role of being a student (Medrano et al., 2014). Various applications of the scale have produced scores with a high level of reliability, with Cronbach’s alpha coefficients falling between 0.86 and 0.94 (Lent et al., 2005, 2007). Application of the scale in Argentina (Medrano et al., 2014) and Chile (Vergara-Morales et al., 2018) demonstrate adequate construct validity, yet in both cases there were large numbers of female students, as in the original version. The two Spanish versions of the AS scale (Medrano et al., 2014; Vergara-Morales et al., 2018) adapted the original AS scale, modifying items 1, 2, 5, and 6 to contextualize the measurement with a university degree. The Likert scale was modified in the original, offering 7 response options from only 1 (completely disagree) to 7 (completely agree). All adaptations of the scale found methodological issues depending on the country of application. This implies it could be useful in translating and validating the scale for the Spanish university population with sex and age invariance: this also ensures that the evaluation instrument is measuring the same construct, regardless of sex or age (Cheung and Rensvold, 2002).

The AS scale correlates with SE, academic persistence, and life satisfaction (Lent et al., 2007: SE is a domain specific, social-cognitive variable, which can predict overall satisfaction (Gallagher et al., 2020): It is future-oriented with goal-directed behavior. Moreover, it involves how one can perform specific behaviors to achieve a desired outcome. SE emphasizes personal agency, or one’s influence over events. While it includes a perceived capacity to perform the actions to achieve a certain goal, it does not emphasize intention or determination, as would be the case with agency thinking, or the ability to produce the steps to accomplish a goal, the case with pathway thinking (Rand, 2017): it is also related to EI (Morales-Rodríguez and Pérez-Mármol, 2019).

On the other hand, cooperative learning (CL) is understood as an instructional approach based on teamwork and builds knowledge, SE, and the acquisition of competence and social skills; it is also associated with the development of students’ cognitive, metacognitive, and motivational skills, which promote self-regulated learning (Fernandez-Rio et al., 2017). Early studies on CL emerged in the 1960s (Slavin, 2011), which are now being studied while focusing on work techniques, academic performance, and its link with affective and social variables; this acts as a positive methodology for students. As a learning technique, CL is probably the best-documented an instructional approach, the one with the most research (Trianes, 2014). CL’s benefits in higher education have been confirmed in numerous studies (García et al., 2001; Torrego, 2019) and are characterized by its link to SE, increased social skills, supporting others, and autonomous group interaction. CL enhances the development of strategies that encourage teachers to create dynamic classes and promote university and high-school participation (Fernández-Espínola et al., 2020). We must understand if modifying university courses toward this instructional approach will improve students’ AS—as they have a special interest in cooperative work, using a team value, one of the relevant motivational variables in group SE (León del Barco et al., 2017), as well as effective when learning objectives are intended for students’ social development.

This research consists of two studies. The aim of Study 1 is to evaluate the psychometric properties of AS, with differential item functioning and invariance of the measure by sex or age. The aim of Study 2 is to determine whether AS improves following a teaching methodology based on CL. This effort tests whether SE and EI predict AS, based on CL in students.

## Method study 1

### Participants

The Study 1 sample consisted of 284 participants (see Table 1), in which 146–51.41% were female and 138–48.59% were male, as well as 18–23-years-old (*M* = 20.8, *SD* = 1.36).

**TABLE 1:** Sociodemographic data for the Study 1 sample.

	N (%)	ξ^2^	d.f
Sex		6.75^ns^	1
Women	146 (51.41)		
Men	138 (48.59)		
Age		2.48^ns^	2
18–19	93 (32.75)		
20–21	96 (33.80)		
22–23	95 (33.45)		
Population of subjects’ place of residence		3.12^ns^	3
<5.000	58 (20.42)		
5,000–49,999	86 (30.28)		
50,000–100,000	85 (29.93)		
>100,000	55 (19.37)		
Total	284 (100)		

ξ^2^, Chi-Square; *p < 0.05; **p < 0.01; ns, Not significant; d.f., degrees of freedom.

### Instruments

#### Sociodemographic data sheet

We prepared a fact sheet for this study to encapsulate information on sex, age, and location.

AS from Lent et al. (2005). This is a seven-item scale that evaluates the level of AS through a Likert-type response from 1 (strongly disagree) to 5 (strongly agree), where the higher the score, the higher the AS. It demonstrates a unidimensional structure and a high level of internal consistency via alpha (0.90) in university students (Lent et al., 2005).

*Positive Mental Health (PMH)* from Lukat et al. (2016) measures positive psychosocial wellbeing, where the higher the score, the more positive the mental health. This unidimensional scale is made up of 9 items with a 4-point Likert-type response (e.g., “I enjoy my life:” 0 = I disagree up to 3 = I agree). It demonstrates high levels of reliability in the original version for university students and the general population of Germany (alpha = 0.92, alpha = 0.93). In this study, we found an alpha of 0.96.

### Procedure

First, we requested approval of the study by the Research Ethics Committee of the University of Jaen, and sought permission to adapt the original author’s scale for AS. We asked two outside bilingual translators (English-Spanish) to produce a Spanish translation of AS. This translation was subsequently revised and translated into English by another bilingual student with a Ph.D in Psychology, who had no connection to this study, but who made appropriate adjustments in the terminology: there had been disagreement with previous translators, and then the final version of the instrument was produced in Spanish (AS-S). We followed all guidelines for adaptation of evaluation instruments in psychology (Muñiz et al., 2013; Muñiz and Fonseca-Pedrero, 2019).

The instrument maintained a Likert-type scale ranging from 1 (strongly disagree) to 5 (strongly agree).

### Ethical considerations

Participants completed the informed consent and questionnaires in Spanish. The Ethics Committee of the University of Jaen approved the study (Code: MAR.20/15.PRY), following guidelines of the Declaration of Helsinki (World Medical Association, 2013). Participation was voluntary and subjects could withdraw at any time. All data were treated per the UE Regulation 2016/679 of the European Parliament and the Council of 27 April 2016, for both Personal Data and The Organic Law 3/2018 of 5 December, related to a guarantee of digital rights.

### Data analysis

Missing data accounted for less than 1%, and the Hot-Deck Multiple-Inputation method for network inference was applied (Lorenzo-Seva and Van-Ginkel, 2016). We first produced a descriptive analysis of the items and performed a confirmatory factor analysis (CFA) using SPSS 23 AMOS (IBM Corporation, 2013) to confirm the structure of AS. The CFA was generalized least squares (GLS) due to multivariate non-normality and sample size (Olsson et al., 2000; Byrne, 2010; Ferrando et al., 2022). The fit indices were the χ^2^/df ratio, the root mean square error of approximation (RMSEA), the Comparative Fit Index (CFI), and the Tucker-Lewis Index (TLI). The goodness-of-fit of the model was considered satisfactory when the TLI and CFI ≥ 0.95, and the RMSEA approached 0.06 (Kline, 2016). We also analyzed whether there were differences in the invariance of the measure by sex or age, using multi-group CFA with AMOS. We specified two nested models for sex and three for age. A configural invariance analysis (reference model) was able to check whether the groups (gender and age) associated the same subsets of items with the construct. Metric invariance was analyzed to check if the factor loadings between each item and the factor itself were the same in all groups. Scalar invariance could assess if the differences between groups, indicated by the items, were the same for all items (Cheung and Rensvold, 2002; Van de Schoot et al., 2012). We used the Satorra-Bentler scale (χ^2^) and its *p*-values, along with RMSEA with 90% CI and CFI for invariance of the measure as an incremental adjustment index (Hooper et al., 2008). There is invariance of the measure when *p* > 0.05 for Δχ^2^ (considering sample size bias); RMSEA ≤ 0.05 and the ΔCFI value of the models compared is < 0.01 (Byrne, 2016). We used descriptive analyses of the scale, with convergent validity measured by calculating the Pearson correlation coefficient with the PMH scale, and reliability measured by means of internal consistency (Cronbach’s alpha and McDonald’s omega coefficients). Statistical significance required all tests to be at a minimum of *p* < 0.05.

## Results study 1

Table 1 presents the sociodemographic data of this sample.

The mean scores of the AS items were higher than the theoretical midpoint of the scale (i.e., 2). The lowest mean was in item 1 (*M* = 3.20; *SD* = 0.92). The correlation between item and total was low and negative for item 1 (–0.26). Reliability of internal consistency, estimated by the ordinal alpha, was 0.70 for the total sample; this improved with the removal of element 1 (Table 2); we maintained it in the confirmatory factor analysis (Table 3).

**TABLE 2:** Descriptive statistics, skewness and kurtosis indices, and item analysis for AS.

	*M* (*SD*)	*K-S*	S	K	*r* Item-total	α If item deleted
					
			SE (0.09)	SE (0.17)		
Item 1	3.20 (0.92)	0.29**	–0.88	–0.26	–0.16	0.79
Item 2	4.02 (0.91)	0.30**	–0.45	0.30	0.76	0.44
Item 3	4.57 (0.57)	0.39**	–1.57	–2.11	0.58	0.51
Item 4	3.90 (0.67)	0.26**	–0.36	–0.19	0.64	0.49
Item 5	4.95 (0.56)	0.33**	–1.34	1.29	0.61	0.54
Item 6	4.20 (0.59)	0.26**	–0.85	0.11	0.56	0.53
Item 7	4.29 (0.60)	0.28**	–0.97	0.29	0.64	0.52
Total	30.73 (6.25)	0.09**	–0.82	0.63	1	0.70

M, Mean; SD, Standard deviation; S, Skewness; K, Kurtosis; SE, Standard error of skewness and kurtosis; K-S, Kolmogorov-Smirnov test.

*Significant correlation at the 0.05 level (bilateral).

**Significant correlation at the 0.01 level (bilateral).

**TABLE 3:** Goodness-of-fit indices for AS (original version and version with item 1 removed) in Spanish university students.

	χ^2^	df	χ^2^/df	*P*	RMSEA (95% CI)	CFI	TLI	RMR	GFI
Model 1	118.42	21	3.11	0.00	0.05 [0.03;0.09]	0.91	0.83	0.07	0.71
Model 2	61.34	19	1.97	0.00	0.02 [0.01;0.03]	0.96	0.97	0.02	0.92

Model 1, Confirmatory factor analysis of seven items (AS original version); Model 2, Confirmatory factor analysis with six items (AS with item 1 removed); χ^2^, Chi-square; df, degrees of freedom; χ^2^/df, Chi-square goodness-of-fit index; p, significance level; RMSEA, Root mean square error of approximation; CFI, Comparative Fit Index; TLI, Tucker-Lewis Index; RMR, Root Mean Residual; GFI, Gamma Index.

### Confirmatory factor analysis

**FIGURE 1:**
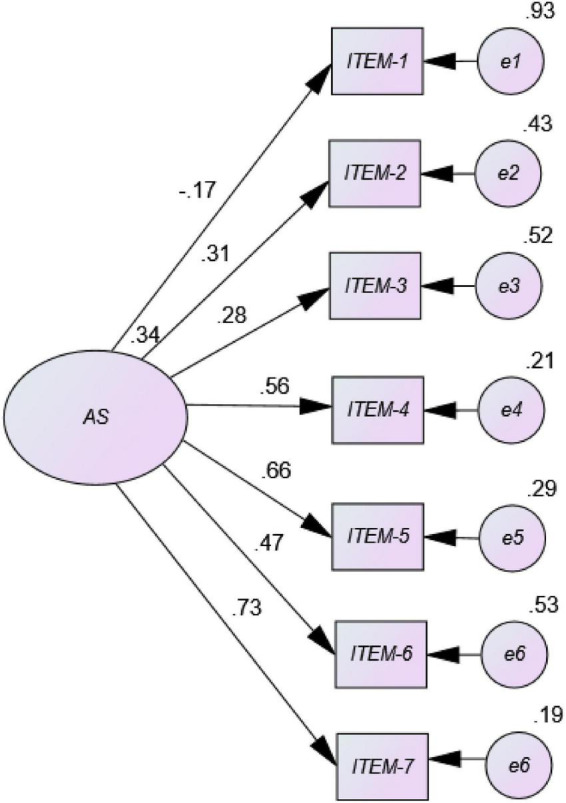
Path diagram of the unidimensional model corresponding to AS in Spanish university students (Model 1 = Original version).

**FIGURE 2:**
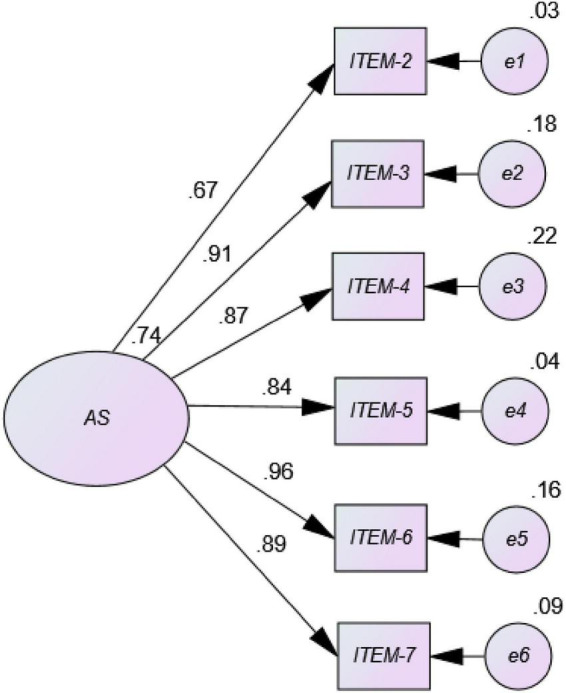
Path diagram of the unidimensional model corresponding to AS in Spanish university students (Model 2 = eliminating item 1).

The results from the univariate and multivariate normality analyses showed there was neither univariate nor multivariate normality in the item distribution in this sample (Mardia = 731.32; Mardia, 1970). Figure 1 (AS original version) shows a negative factorial load for item 1 with other items presenting factorial loads below 0.50. However, Figure 2 (eliminating item 1) shows high factorial loads (> 0.50) for most items in the AS path diagram and values of the normalized loads (coefficients from β) ranging from a minimum of 0.67 for item 2, to a maximum of 0.96 for item 6. These results indicate acceptability and goodness of fit of a six-item Model 2, as data confirm a unidimensional structure with 6 elements in a sample of university Spanish students.

### Measurement invariance

Table 4 yields results of measurement invariance, showing that the CFA models specified for men and women and each age group demonstrated a good fit, indicating that a multiple group CFA was appropriate. The metric invariance and scalar invariance by sex showed that men and women understood the AS-Spanish items (AS-S) (Appendix Table A1) in the same way, demonstrating good levels of fit [Δχ^2^_(1)_ = 1.11; *p* > 0.05; Δχ^2^_(1)_ = 1.08; *p* > 0.05]. The comparison of groups by age showed there was no variation in AS-S according to age brackets (Δχ^2^_(2)_ = 1.16; *p* > 0.05; Δχ^2^_(2)_ = 1.22; *p* > 0.05).

**TABLE 4:** Indices of fit for invariance tests by sex and age.

	χ^2^	df	χ^2^/df	p	RMSEA (95% CI)	CFI	Δχ^2^	ΔCFI
Men (*n* = 138)	48.11	26	1.85	0.05	0.03 [0.01; 0.04]	0.96		
Women (*n* = 146)	51.18	26	1.54	0.00	0.02 [0.01; 0.03]	0.97		
Configural invariance sex	88.54	45	2.01	0.01	0.03 [0.02; 0.03]	0.99		
Metric invariance sex	89.16	45	1.98	0.01	0.03 [0.03; 0.04]	0.98	1.11^ns^ (Δdf = 2)	0.01
Scalar invariance sex	95.11	55	1.72	0.03	0.03 [0.02; 0.04]	0.98	1.08^ns^ (Δdf = 2)	0.01
Age (18–19)	118.20	51	2.21	0.00	0.01 [0.01; 0.03]	0.97		
Age (20–21)	146.23	53	2.54	0.00	0.03 [0.02; 0.04]	0.96		
Age (22–23)	157.49	68	2.14	0.01	0.03 [0.01; 0.04]	0.96		
Configural invariance age	187.65	71	1.89	0.02	0.02 [0.02; 0.03]	0.95		
Metric invariance age	187.78	98	1.91	0.02	02 [0.01; 0.03]	0.98	1.16^ns^ (Δdf = 1)	0.01
Scalar invariance age	195.29	99	1.97	0.03	04 [0.03; 0.04]	0.99	1.22^ns^ (Δdf = 2)	0.02

χ^2^, Chi-square; d.f., degrees of freedom, p, significance level; RMSEA, Root mean square error of approximation; CFI, Comparative Fit Index; Δχ^2^, Difference test between the configural and metric or scalar invariance models; ΔCFI, Difference test between Comparative Fit Index.

*p < 0.05; **p < 0.01; ns, not significant.

### Reliability and convergent validity

The results for internal consistency (Cronbach’s alpha and the Omega coefficient) were adequate, with a strong positive correlation between AS-S and positive mental health, confirming its convergent validity (Table 5).

**TABLE 5:** Descriptive statistics, reliability, and convergent validity.

AS-S	M(SD)	Min.	Max.	Ω	A	*r*_*PMH*_
	72.09 (3.10)	40	120	0.89	0.91	0.98

AS-S, Academic satisfaction scale (Spanish version); M, Mean; SD, Standard deviation; ω, Omega coefficient; α, Cronbach’s Alpha; r_PMH_, Correlation with positive mental health (PMH).

## Method study 2

### Participants

Study 2 consisted of 261 undergraduate students for 2 years of the psychology degree program. Almost three quarters (186; 71.26%) were female, and 75 (28.74%) were male. We randomly divided the sample into a control group (CG) and an experimental group (EG) (Mondo et al., 2021). Table 6 shows its sociodemographic data.

**TABLE 6:** Sociodemographic data for the sample in Study 2.

	N (%)	CG	EG	*T*	d.f.
Sex				0.02^ns^	259
Women	186 (71.26)	91 (48.92)	95 (51.08)		
Men	75 (28.74)	36 (48)	39 (52)		
Age				0.07^ns^	259
18–19	71 (27.20)	33 (46.48)	38 (53.52)		
20–21	89 (34.10)	45 (50.56)	44 (49.44)		
22–23	101 (38.70)	49 (48.51)	52 (51.54)		
Number of inhabitants and place of residence				2.37^ns^	259
<5.000	74 (28.35)	35 (47.30)	39 (52.70)		
5,000–49,999	79 (30.27)	38 (48.10)	41 (51.90)		
50,000–100,000	61 (23.37)	32 (52.46)	29 (47.54)		
>100,000	47 (18.01)	22 (46.81)	25 (53.19)		
Total	261 (100)	127 (100)	134 (100)		

CG, Control Group; EG, Experimental Group; t, Student-T.

*p < 0.05; **p < 0.01; ns, not significant; d.f., degrees of freedom.

### Instruments

#### Academic satisfaction

The original scale is developed by Lent et al. (2005). This was adapted to Spanish in Study 1. It is a scale with six elements that assess the level of AS through a Likert-type response from 1 (strongly disagree) to 5 (strongly agree), where the higher the score, the higher the AS. It demonstrated a unidimensional structure, high levels of internal consistency (ω = 0.89; α = 0.91), and a strong correlation with PMH (*r* = 0.98) in Spanish university students.

#### General self-efficacy scale-GSE

The self-efficacy scale was created by Schwarzer and Jerusalem (1995), then Bäßler and Schwarzer (1996) translated it into Spanish in 1996, but the psychometric properties in the general Spanish population are from Sanjuán et al. (2000). It measures general SE, the belief that one’s actions create successful outcomes, and is made up of 10 items with a scale from 1 (not at all true) to 4 (completely true). There are no cut-off points, scores vary from 10 to 40 points, and the higher the score, the greater the overall perceived SE. Internal consistency of the Spanish version was 0.84—while in this study, Cronbach’s alpha was 0.93.

#### Wong-law emotional intelligence scale

The original scale is developed by Wong and Law (2002). We used the Spanish version from Extremera et al. (2019) for university students. This scale consists of 16 items that measure four aspects of EI: assessment of one’s own emotions (EAE), assessment of other people’s emotions (OEA), use of emotion (UOE), and regulation of emotion (ROE). The instrument uses a seven-point Likert-type response, ranging from 1 (completely disagree) to 7 (completely agree). The reliability of the Spanish version via Cronbach’s alpha was excellent for the total scale (α = 0.91) with subscales demonstrating satisfactory internal consistency (Cronbach’s α ranged from 0.79 to 0.84). In this study, the reliability through Cronbach’s alpha was 0.90.

### Procedure

We adapted a test for AS for Spanish university students (Study 1) (Appendix Table A1). Then we used a conventional teaching methodology at the beginning of practical classes for students in psychology degree subjects for both experimental and CGs. The methodology wanted the teacher to explain the practical activity, so students in the subsequent session could ask questions which had been resolved. Halfway through the practical sessions, the teachers changed to a CL-based instructional approach in the EG, while the CG continued unchanged. We applied evaluation tests at the beginning and end of the process.

### Ethical considerations

This study follows the guidelines of the Declaration of Helsinki (World Medical Association, 2013) and the Ethics Committee of the University of Jaen approved the study (Code: MAR.20/15.PRY).

### Data analysis

We began by analyzing differences in all the psychosocial variables and the result variable (AS) between the two time points using the parametric Student *t*-test. We also calculated indices of statistical power and effect size; this refers to the magnitude of the differences found in the study, and statistical power refers to the level of validity of the findings (Cohen, 1988; Grissom and Kim, 2012). Following that, we performed a hierarchical regression analysis to determine which sociodemographic and psychosocial variables could predict higher levels of AS in university students in the EG. The level of statistical significance for all tests was a minimum of *p* < 0.05. Statistical analyses used SPSS version 22.0 (IBM Corporation, 2013), and the statistical power and effect sizes were determined using G*Power 3.1.9.7 (Faul et al., 2009). According to Cohen (1988), when *d* = 0.2, the effect size is small, when it is 0.5, the effect size is moderate, and when it is 0.8, the effect size is large.

## Results study 2

Results of the comparison (*t*) between time points for both groups (control and experimental) (Table 7) showed that there were notable differences in mean scores for AS in the EG but not in the CG. This was particularly notable in the scale total [*t*_(259)_ = 9.12; *p* < 0.01; (8.27–10.02)]. We also saw differences in SE [*t*_(259)_ = 8.61; *p* < 0.01; (7.11–9.26)], but not in EI [*t*_(259)_ = 4.32; *p* > 0.05; (3.75–4.89)] post-training. The effect size was large, while the statistical power was high for AS (*d* = 0.92; Pow = 97) and other variables.

**TABLE 7:** Differences in academic satisfaction between two time points for both groups (Study 2).

	CG		EG						
						
	Pre *M* (*SD*)	Post *M* (*SD*)	Pre *M* (*SD*)	Post *M* (*SD*)	*T*	95%CI (*t)*		*d*	Pow
								
						L.L	U.L.		
AS	19.32 (1.77)	19.37 (1.49)	19.67 (2.93)	29.91 (1.78)	9.12**	8.27	10.02	0.92	0.97
SE	26.98 (1.21)	25.18 (1.56)	27.53 (2.45)	36.97 (2.66)	8.61**	7.11	9.26	0.91	0.88
EI	65.02 (4.35)	64.89 (4.42)	64.98 (4.35)	65.11 (4.78)	4.32^ns^	3.75	4.89	0.89	0.86

AS, Academic satisfaction; SE, Self-Efficacy; IE, Emotional Intelligence; M, Mean; SD, standard deviation; t, post-comparison.

*p < 05; **p < 0.01; ns, not significant; d, effect size; Pow, statistical power (1-β).

Initially, the criteria for suitability and goodness of fit of the blocks for each model were partially met. In particular, the independence of errors through the Durbin-Watson test indicated that this assumption of suitability was only met in the outcome variable AS in model three (DW_block1_ = 0.04; DW_block2_ = 0.05; DW_block3_ = 1.95) (Yoo et al., 2014). The assumption of non-multicollinearity was also inadequate for AS in two of the three predictive blocks, as the value was below 5 (Kleinbaum et al., 1988) (Variance Inflation Factor—VIF_block1_ = 7.65; VIF_block2_ = 5.96; VIF_block3_ = 1.95). The hierarchical regression applying to block 3 showed that sociodemographic and psychological variables predicted a higher level of AS in a sub-sample of Spanish university students (Table 8). Block 3 (set of independent variables) was significant and explained 91.7% of resilience [*R*^2adj^ = 0.917; *F*_(1.261)_ = 3169.12; *p* < 0.01] (Table 8). This block indicates variables (sociodemographic and psychological) that predict a high level of AS: Ages 22–23 [β = 9.11; (95%) CI = 8.22–9.98; *p* < 0.01], with a high level of SE [β = 8.62; (95%) CI = 8.11–8.29; *p* < 0.01], and high emotional regulation [β = 7.96; (95%) CI = 6.45–8.13; *p* < 0.01].

**TABLE 8:** Hierarchical prediction models of sociodemographic and psychological variables in Study 2 (EG = 134).

Block and variables	*R* ^2adj^	SE	*F*	*t*	β	CI (95%) (β)	
						
						L.L.	U.L.
1	0.254	6.98	17.65 ^ns^	0.32*^ns^*			
Sex (women)					0.51	–0.22	2.78
Age (22–23)					0.27	–0.11	1.92
2	0.721	4.46	129.11**	2.18*			
Sex (women)					2.22	–0.12	3.59
Age (22–23)					0.23	–0.08	0.89
Number of inhabitants (<5.000)					1.89	0.31	2.19
Self-efficacy					1.27	0.48	1.22
3	0.917	0.89	3169.12***	53.45***			
Age (22–23)					9.11	8.22	9.98
Self-efficacy					8.62	8.11	8.29
EI (assessment of other people’s emotions-OEA)					5.27	4.22	6.46
EI (regulation of emotion-ROE)					7.96	6.45	8.13

R^2adj^, Adjusted R-square; SE, standard error; F, test statistic (ANOVA).

*p < 0.05; **p < 0.01; ***p < 0.001.

ns, non-significant; t, predictive variable test statistic; β, result of regression or beta equation; 95%CI, confidence intervals; LL, lower limit; UL, upper limit; EI, emotional intelligence.

## Discussion

The aim of Study 1 was to evaluate the psychometric properties of AS, and differential function of items, with invariance by sex or age in university students.

Despite authors who studied AS, such as Medrano et al. (2014), and relating it to student wellbeing, previous measures were not sought for AS focused on psychological wellbeing: In a university context, it is positively related to quality of learning, diversifying instruments with adequate psychometric properties as a good measure for this variable. Methodological problems may be conditioned by the existence of bias in the responses, as evidenced by different levels of error, that would probably be linked to response error if they had been described in another way.

Results from the EFA support the single-factor structure of the AS scale, although with the removal of item 1. This item measures satisfaction, although in the general realm of psychology, adapting it to a university course after being translated signified that it lost some meaning. There is a difference in specializing in psychology vs. choosing a specific subfield, indicating that the translation by Vergara-Morales et al. (2018) was unsuitable for a university setting (item 1: I am satisfied with the decision to take this subject), as neither of the subjects was optional, but the translation implied they were.

In addition, we determined that the results had good reliability in the Spanish higher education context, with adequate scores for internal consistency and a good relationship with the theoretical construct of AS. We can conclude that the single-factor solution is consistent compared to the original model, considering the adaptation of 6 items (AS-S) to a Spanish context. We were unable to replicate the original factor structure, as it is likely that issues with the measurement model fit were due to biases in the questionnaires for item 1, as it did not discriminate appropriately.

None of the studies in the literature had examined the age or sex invariance of AS, and no studies found differences in it due to these variables. Our results for measurement variance, with the CFA models for men and women as well as for each age group demonstrating a good fit, found that a multigroup CFA was appropriate. The invariance of measurement by sex demonstrated that men and women understood the items in the AS-S scale in the same way, as it found no variation in AS responses due to the age brackets presented. We conclude that there is a measurement of invariance in terms of age and sex in this specific population of Spanish university students.

The results of Study 1 resolved what might have been a significant issue for the validity of Study 2. Many more women took part in Study 2 than men, which is a common limitation in psychology research, as female participation tends to be greater than that of males. In addition, more women use psychology degrees than men. This might be because women are more likely to deal with problems and ask for help (Liddon et al., 2017). This is no longer a limitation, as Study 1 showed that men and women had similar understandings of the items in our satisfaction scale.

In Study 2, we aimed to determine whether the CL improved student satisfaction. We found that satisfaction increased significantly and that there were no differences between the two test time points in either SE or EI. In addition, the more satisfied students were those with higher levels of SE, who scored higher in emotional regulation (within EI); this is consistent with studies linking these variables together (Fernandez-Rio et al., 2017; Morales-Rodríguez and Pérez-Mármol, 2019).

Consequently, we can now realize the clinical benefits of these findings. The results indicate that university students who are more satisfied with the implementation of CL have higher self-efficacy and greater emotional regulation, so it seems that the change in the way of carrying out academic practice in the subjects has benefited them.

### Limitations and future research

One study limitation is the convenience sampling method we used, which limits the generalizability of the findings. Future studies must consider a more representative sample. The division into experimental and CGs in Study 2 presents problems of causal interpretation regarding the manipulation. However, we believe it is a way of comparing the variability in a methodology that would otherwise have been more difficult. Additional aspects of validity (e.g., local independence between items; rating scale threshold disorder) should also be considered before concluding about the validity evidence of a given test. There are other models in modern test theory that could have been used to explore the data further. However, given our review of previous studies, and including findings from this study, methodological analysis seems to influence the results of a validity study, and must be included in the interpretation of empirical findings. The integration of theoretical implications of a construct has methodological assumptions in our data analysis, including empirical findings from diverse populations and groups; this is a complex, critical process that must be monitored consistently to support quality control in instrument development.

## Conclusion

A reduced 6-item AS scale from the study sample demonstrates better psychometric properties (unidimensionality) and a similar level of precision to the original 7-item scale. The original AS was translated into Spanish and tested for reliability and validity with a convenience sample. To our knowledge, this is the first study attempting to assess these variables, exploring structural characteristics and confirming the most appropriate structure in the sample. We examined invariance by sex and age. The AS-S demonstrated good internal consistency and met requirements for psychometric properties. The practical implication of this study is that AS-S is a reliable instrument to assess AS in men and women ages 18–23. This study shows that student satisfaction increased after CL, given the circumstances of this study.

## Data availability statement

The data analyzed in this study is subject to the following licenses/restrictions: The data that support the findings of this study are available from the corresponding author upon reasonable request. Requests to access these datasets should be directed to MR-B, marobles@ujaen.es.

## Ethics statement

The studies involving human participants were reviewed and approved by University of Jaen (Spain): Code: MAR.20/15.PRY. The patients/participants provided their written informed consent to participate in this study.

## Author contributions

MR-B and DS-T: conceptualization, data acquisition, data management, data analysis, data interpretation, manuscript preparation, and writing – original draft preparation. ÓG and AG: writing – original draft preparation, critical review, commentary, and revision. JC: conceptualization, data acquisition, writing, critical review, commentary, revision, and editing. All authors contributed to the article and approved the submitted version.

## Appendix

**TABLE A1:** Academic satisfaction scale for Spanish university students (AS-Spanish).

Consider the following statements: Please mark an X through the number in each row that represents your opinion	Completely disagree	Disagree	Neither agree nor disagree	Agree	Completely agree
1. I feel comfortable with the atmosphere created in this subject/Me siento cómodo o cómoda con el ambiente creado en esta asignatura	1	2	3	4	5
2. For the most part, I have enjoyed my work in this subject/En su mayor parte, estoy disfrutando o he disfrutado de mi trabajo en esta asignatura	1	2	3	4	5
3. In general, I am satisfied with this subject/En general estoy satisfecho o satisfecha con esta asignatura	1	2	3	4	5
4. I enjoy the level of intellectual stimulation this subject yields/Disfruto del nivel de estimulación intelectual que me proporciona o ha proporcionado esta asignatura	1	2	3	4	5
5. The subject content interests me/Me entusiasman los contenidos transmitidos en esta asignatura	1	2	3	4	5
6. I like what I am learning or have learned in this subject/Me gusta lo que estoy aprendiendo o he aprendido en esta asignatura	1	2	3	4	5
